# A New Player in the Mechanobiology of Deep Fascia: Yes-Associated Protein (YAP)

**DOI:** 10.3390/ijms242015389

**Published:** 2023-10-20

**Authors:** Carmelo Pirri, Brasilina Caroccia, Andrea Angelini, Maria Piazza, Lucia Petrelli, Ilaria Caputo, Chiara Montemurro, Pietro Ruggieri, Raffaele De Caro, Carla Stecco

**Affiliations:** 1 Department of Neurosciences, Institute of Human Anatomy, University of Padova, 35121 Padova, Italy; rdecaro@unipd.it (R.D.C.); carla.stecco@unipd.it (C.S.); 2 Department of Medicine-DIMED, University of Padova, 35128 Padova, Italy; brasilina.caroccia@unipd.it (B.C.); maria.piazza@unipd.it (M.P.); lucia.petrelli@unipd.it (L.P.); ilaria.caputo@unipd.it (I.C.); 3 Department of Orthopedics and Orthopedic Oncology, University of Padova, 35128 Padova, Italy; andrea.angelini@unipd.it (A.A.); pietro.ruggieri@unipd.it (P.R.); 4 Independent Researcher, ty Padova, 35120 Padova, Italy; chiaramontemurro220@gmail.com

**Keywords:** fascia, thoracolumbar fascia, extracellular matrix, fibroblasts, Yes-associated protein (YAP), remodeling

## Abstract

Recent studies have demonstrated that fascial fibroblasts are susceptible to mechanical stimuli, leading to the remodeling of the extracellular matrix (ECM). Moreover, the extensive literature on Yes-associated protein (YAP) has shown its role in cell mechanics, linking cell properties, such as shape, adhesion, and size, to the expression of specific genes. The aim of this study was to investigate the presence of YAP in deep fascia and its activation after a mechanical stimulus was induced via a focal extracorporeal shockwave (fESW) treatment. Thoracolumbar fascia (TLF) samples were collected from eight patients (age: 30–70 years; four males and four females) who had undergone spine elective surgical procedures at the Orthopedic Clinic of University of Padova. YAP was measured in both tissue and TLF-derived fibroblasts through immunoblotting. *COL1A1* and *HABP2* gene expression were also evaluated in fibroblasts 2, 24, and 48 h after the fESW treatment. YAP was expressed in all the examined tissues. The ratio between the active/inactive forms (YAP/p-YAP) of the protein significantly increased in fascial fibroblasts after mechanical stimulation compared to untreated cells (*p* = 0.0022). Furthermore, *COL1A1* and *HABP2* gene expression levels were increased upon treatment. These findings demonstrate that YAP is expressed in the deep fascia of the thoracolumbar region, suggesting its involvement in fascial mechanotransduction processes, remodeling, regeneration, and fibrogenesis. This study indicates, for the first time, that YAP is a “new player” in the mechanobiology of deep fascia.

## 1. Introduction

An increasing number of studies has demonstrated the role of deep fascia in pain, proprioception, motor coordination, and tissue biomechanics [1,2,3]. Although it is generally accepted that deep fasciae, forming a ubiquitous network throughout the whole body, have a passive role in musculoskeletal biomechanics [4], it is undeniable that several mechanical forces, such as shear stress, pressure, and tensional forces, influence their function. Fascial tissue has a complex architecture due to the presence of different cell types, namely fibroblasts, myofibroblasts, fasciacytes, and adipocytes. Fascial behavior is the result of internal pulling forces, determined by the tension and organization of the cell cytoskeleton, and external forces, exerted by the surrounding extracellular matrix (ECM) [5]. These mechanical forces promote the activation of several signaling pathways that allow the cells to modify their shape, position, and function in response to the surrounding environment [6].

The main cell population found in the fascial tissue are fibroblasts [7], that under physiological conditions, are in their resting state with a long shuttle-shaped or flattened triangular cell body. Fibroblasts are critical in shaping and in maintaining fascial tissue organization and integrity by secreting precursors of the ECM. The ECM is a highly dynamic matrix composed of collagen, proteoglycans/glycosaminoglycans, elastin, laminin, and other glycoproteins, in which cells interact and perform their functions [5,8]. Upon stimulation, resting-state fibroblasts are activated, acquiring distinct phenotypic features, from spindle-shaped to stellate-shaped activated fibroblasts with a higher contractile or synthetic phenotype accompanied by an increase in cell size and deposition of the ECM [8,9,10]. Recently, Wang T et al. investigated cellular heterogeneity in the deep fascia of patients with acute compartment syndrome via single-cell RNA sequencing. They identified five subclusters of fibroblasts in the examined tissues that underline inter- and intra-tissue heterogeneity amongst fibroblasts, as already reported for other tissue and organs [8,11].

The processes through which cells, tissues, and organs can sense and respond to mechanical cues to regulate numerous biological processes, including development, differentiation, physiology, and diseases is known as mechanosensing [12]. Cells interacting with the microenvironment and between themselves exert mechanical forces that are converted into biochemical signals that affect cell morphology, cytoskeletal organization, survival, proliferation, differentiation, and gene expression. These processes involve both intracellular and extracellular components, such as integrins, ECM proteins, and the cytoskeleton. The mechanotransduction mechanisms through which mechanical signals are transduced into a cascade of biochemical events are challenging to clarify. Focal extracorporeal shockwaves (fESWs) are sound waves commonly used to mimic mechanical stimuli in fibroblasts [13].

Recently, Pirri et al. demonstrated that fascial fibroblasts respond to biomechanical stimuli, promoting ECM remodeling through the deposition of type I and III collagen fibers, along with hyaluronan [14]. Moreover, the sensitivity of fascial fibroblasts to biochemical stimuli has been demonstrated in response to endocannabinoids, sex hormones, and, possibly, angiotensin peptides [15,16,17].

Stecco et al. showed that fibrosis may occur as a long-term consequence of hyaluronan densification due to an excessive collagen fiber deposition and consequent ECM remodeling, and this mechanism was found also in the deep/muscular fascia [18].

Yes-associated protein (YAP), also known as YAP1, is a cellular transducer of mechanical stimuli, such as matrix stiffness and stretch, promoting the expression of gene targets through the activation of the transcription factor the TEAD PDZ-binding motif (TAZ) [19]. YAP activity can be determined through its subcellular localization, as it is active when present in the nucleus [20]. YAP cellular distribution depends on cell shape, stiffness, and organization of the ECM [21], along with shear stress [22]. YAP activity is mainly regulated through the Hippo pathway; when the Hippo kinase cascade is activated, YAP is phosphorylated and stays in the cytoplasm of the cell in its inactive form. In contrast, when the Hippo pathway is inactive, YAP is dephosphorylated, becomes active, and migrates in the nucleus where it binds with members of the TEAD transcription factor family to regulate the expression of gene targets and promote cell proliferation [23].

Changes in the expression of genes caused by mechanical forces in the deep fascia are still unelucidated. Therefore, the aim of the current study was to investigate the role of YAP in deep fascia gene regulation. In detail, we have evaluated the presence of YAP in thoracolumbar fascia (TLF) tissue and isolated fibroblasts and YAP activation in response to mechanical stimuli, i.e., fESWs, in only fascial fibroblasts.

## 2. Result

### 2.1. Yes-Associated Protein (YAP) in the Thoracolumbar Fascia (TLF)

**Figure 1:**
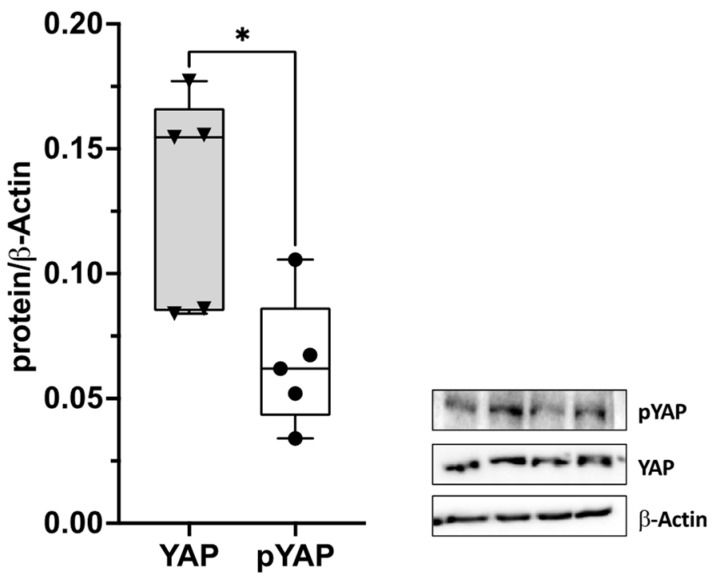
A box and whisker plot displaying densitometric quantifications of YAP and inactive phosphorylated (p)YAP protein levels in TLF tissue. YAP and pYAP were investigated in 5 patients (3 male and 2 female patients, patient n° 2, 3, 5, 7, and 8). On the right, representative blots are shown. β-actin was used as a loading control. * *p* < 0.05.

In order to assess the presence of YAP in deep fascia, the protein expression levels of YAP and inactive YAP, which is phosphorylated at serine 127 (pYAP), were evaluated via immunoblotting in eight TLF tissue samples obtained from four male and four female patients that underwent an elective spine surgical procedure. The relative YAP and pYAP protein levels were normalized to the content of β-actin. We found that both YAP and pYAP were expressed in five tissues (patient n° 2, 3, 5, 7, and 8). Three patients were excluded from the analysis, as the quality and quantity of the protein obtained from the tissue were low (patient n° 2, 4, and 6) (Figure 1).

### 2.2. Fibroblasts Extraction and Characterization

**Figure 2:**
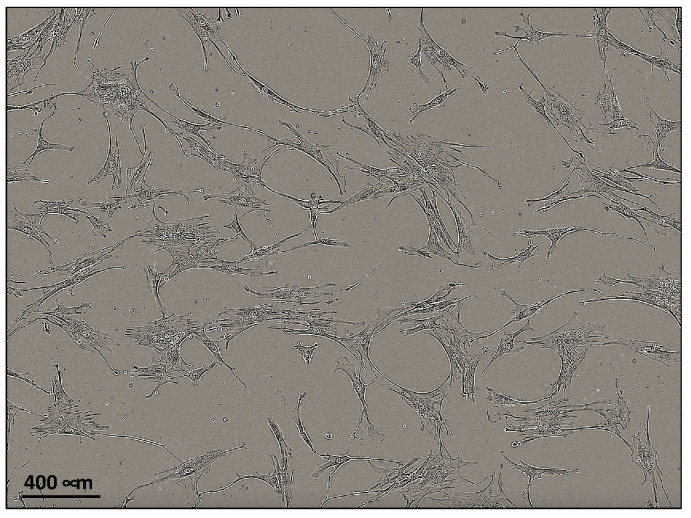
Representative picture showing cultured fibroblasts obtained from the TLF of one male patient (patient n° 3) under a brightfield phase-contrast microscope as they appeared at day 14 of culture (cell passage 1).

Fibroblasts were obtained from TLF of four patients (two male and two female, patient n° 2, 3, 5, and 7) that underwent elective spine surgical procedures. Tissues were minced into pieces <1 mm and cultured at 37 °C and 5% CO_2_ until the point where it was stopped to allow cell isolation and proliferation, as shown in Figure 2 (day 14 of cell culture). The morphology of the cultured cells was spindle-shaped, typical of fibroblasts.

**Figure 3:**
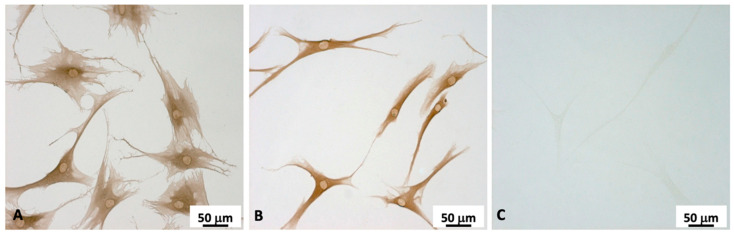
Representative immunocytochemistry for CD90 (panel (**A**)), and vimentin (panel (**B**)) in cells obtained from the TLF tissue of patient n° 3. Cells at passage 3 of the culture were grown on coverslips for 3 days and fixed with 4% PFA. Negative controls were conducted via omission of the primary antibody, confirming the specificity of the immunostaining (panel (**C**)).

We performed immunocytochemistry to verify the expression of the fibroblast markers CD90 and vimentin. CD90, also called Thy1, is a membrane glycoprotein that is expressed on the cell surface and is widely used to identify fibroblasts in the tissue. CD90 is an important regulator of cell–cell and cell–matrix interactions, with significant roles in cellular adhesion and migration, nerve regeneration, and fibrosis [24]. Vimentin, a type III intermediate filament protein, is crucial for fibroblast proliferation, and fibroblasts that are genetically deficient for this protein grow slowly compared to wild-type cells [25]. CD90 (Figure 3, panel A) and vimentin (Figure 3, panel B) immunostaining was evident in all cells, and it was homogeneously distributed throughout the cytoplasm. Both markers also exhibited a perinuclear location. Cell characterizations were performed at passage 3 of the cell culture.

The negative control showed no background staining for the mouse IgG secondary antibody used in our experiments, as no immunosignals were revealed (Figure 3, panel C). 

### 2.3. YAP Activation

**Figure 4:**
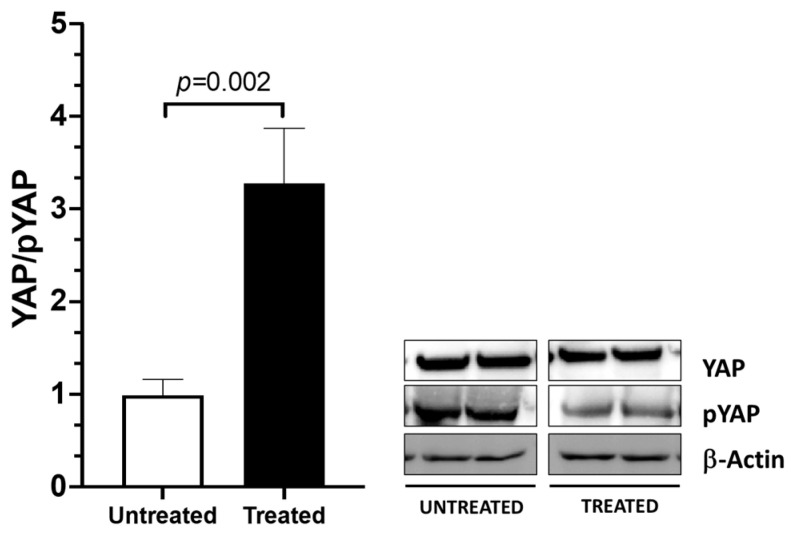
YAP activation was induced through focal extracorporeal shockwaves that mimic a mechanical stimulus. Fibroblasts were obtained from the TLF of 4 patients (2 males and 2 females, patient n° 2, 3, 5, 7), and at passage 5, they were seeded in a 6-well plate and fESWs were applied to the cells. Following this step, 30 min after the fESW treatment, protein extraction was conducted, and total and phosphorylated YAP levels were analyzed via immunoblotting. The ratio of YAP to phosphorylated YAP showed that YAP was activated after 30 min of mechanical stimulus application. Graph bars represent the mean values ± SEM of 4 experiments, each in duplicate.

YAP activity is regulated via S127 phosphorylation; when the protein is phosphorylated, it is inactive and is retained in the cytoplasm of the cell. To clarify whether YAP can be activated in response to mechanical stimuli, fibroblasts, obtained from the TLF of four patients (patient n° 2, 3, 5, and 7) were seeded in a 6-well plate and fESWs were used to mimic mechanical stimuli in vitro. In detail, 30 min after the end of the fESW treatment, protein extraction was performed, and YAP and inactive phosphorylated YAP expression levels were analyzed via immunoblotting. As shown in the bar graph, the ratio between total YAP/inactive YAP (YAP/pYAP) significantly increased in the fascial fibroblasts that were treated with fEWSs compared to the untreated cells (Figure 4).

### 2.4. Collagen Type 1 A (COL1A1) and Hyaluronan-Binding Protein 2 (HABP2) Gene Expression after the fESW Treatment

**Figure 5:**
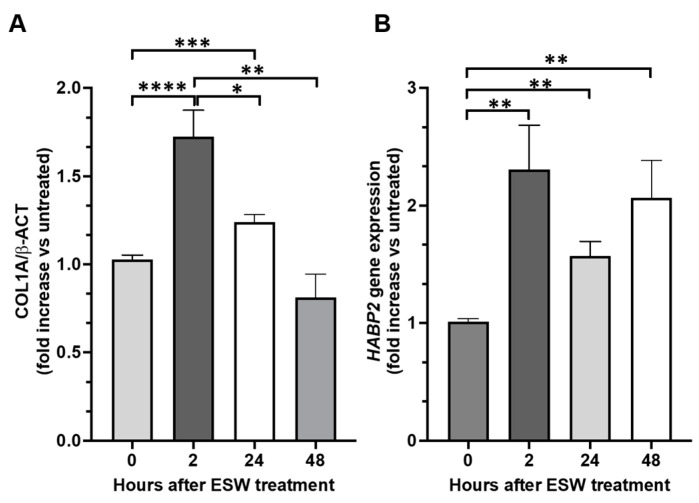
COL1A1 and HABP2 gene expression were investigated in fibroblasts obtained from the TLF of 4 patients (2 males and 2 females) after 2, 24, and 48 h of the fEWS mechanical stimulus via real-time PCR. The treatment enhanced COL1A1 (**A**) after 2 and 24 h, with a peak at 2 h. HABP2 (**B**) gene expression was enhanced after 2, 24, and 48 h of treatment without any differences among them. All data represent the mean ± SD of 4 experiments, each performed in triplicate. * *p* < 0.05 vs. 2 h; ** *p* < 0.01 vs. vehicle; *** *p* < 0.001 vs. vehicle; **** *p* < 0.0001 vs. vehicle.

As YAP activation has been reported to be responsible for the up-regulation of fibrosis-associated gene expression, including of type 1A collagen (COL1A1), we assessed the effect of fEWSs on this gene in fibroblasts obtained from the TLF of patient n° 2, 3, 5, and 7. COL1A1 gene expression was measured 2, 24, and 48 h after the fEWS treatment, and it was significantly enhanced at 2 and 24 h, with a peak at 2 h. After 48 h of treatment, COL1A1 mRNA levels were comparable to those found in untreated cells (Figure 5). We also measured hyaluronan-binding protein 2 (HABP2) gene expression, as a marker of fascial remodeling, after the same treatment and at the same time points. We found that HABP2 gene expression was significantly increased at all examined times without any differences among them.

### 2.5. The fESW Treatment Enhances Fibroblast Proliferation via YAP-Mediated Signaling

**Figure 6:**
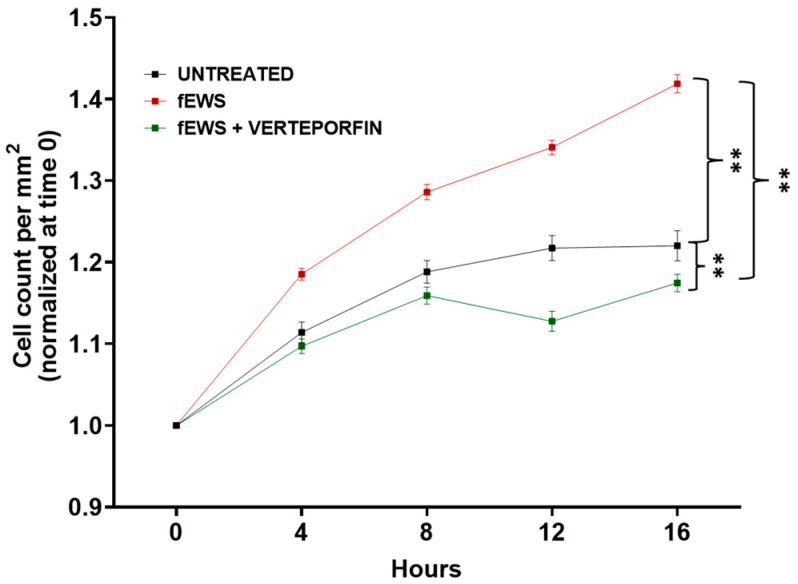
Fibroblast proliferation was measured with the IncuCyte S3 Live-Cell Analysis System. Fibroblasts were obtained from the TLF of 4 patients (2 males and 2 females, patient n° 2, 3, 5, and 7) and were seeded in a 6-well plate and grown until reaching 60% confluence. The black line shows the fibroblast proliferation of untreated cells; in red, is the reported cell proliferation after the fEWS treatment. Prior to the treatment with the fEWSs, the cells were incubated for 1 h with the YAP inhibitor verteporfin (green line). Data represent cell count/mm^2^ (mean ± SEM). ** *p* < 0.0001.

The cell proliferation assay was performed via live-imaging acquisition in the IncuCyte S3 Live-Cell Analysis System, as reported in the Materials and Methods section. We found that fibroblasts treated with fEWSs showed higher proliferation rates compared to non-treated cells (Figure 6). Moreover, the YAP inhibitor verteporfin strongly decreased this effect (*p* < 0.02), showing a lower proliferation rate compared to the cells only treated with fEWSs (Figure 6), suggesting that YAP signaling triggered by fEWSs strongly influences fascial fibroblasts’ proliferation rate.

## 3. Discussion

Mechanosensing is the ability of the cells to sense mechanical stimuli in their microenvironment, and mechanotransduction is the cells’ ability to subsequently translate and reply to mechanical stimuli by programming their behaviors. Many mechanical stimuli can modulate the cells’ behavior, such as ECM stiffness, blood flow, wall or turbulent shear stress, cell shape (geometry), cell density, topographic surface, and cytoskeletal tension. Several models have elucidated and confirmed that YAP acts as a mechanosensor to convey signals that control cell function and biological responses [26,27,28].

To our knowledge, this is the first time that YAP has been described in human fascia. The expression of YAP was investigated with the aid of immunoblotting, showing the existence of both its active and inactive forms in the TLF samples. These data are of main interest, as they reveal a new cellular mechanotransduction mechanism that could play a role in the fasciae remodeling and regeneration processes. To ascertain whether YAP can be activated via mechanical stimuli in the fascial tissue, TLF fibroblasts were treated with fESWs, as previously described by Pirri et al. [14]; this process induced YAP’s dephosphorylation and activation. Our data indicated that the ratio between the two forms of YAP active/inactive (YAP/p-YAP) was statistically different in the treated fascial fibroblasts compared to the untreated (*p* = 0.002).

YAP is a 488 amino acid protein that acts as a transcriptional co-regulator, promoting the expression of genes involved in the proliferation of tissue-specific progenitor cells during tissue renewal and regeneration, as well as, in general, in organ growth [23]. Verteporfin is a small molecule inhibitor of nuclear YAP–TEAD interactions, which acts via the sequestration of YAP in the cytoplasm and promoting YAP degradation [29,30], and it is known to exhibit anti-oncogenic, anti-angiogenic, and anti-proliferative effects [29]. We found that fEWSs strongly influence the fascial fibroblasts’ proliferation rate via YAP signaling, as this event was inhibited by the pre-treatment with verteporfin (Figure 6).

YAP and TAZ are well known for being the effectors of the Hippo signaling cascade [23]; when this pathway is activated, YAP and TAZ enter the nucleus and regulate the expression of specific genes. YAP is a primary sensor of cell structure, shape, adhesion, and polarity, as it is responsible for the transduction of mechanical signals from the tissue architecture and surrounding ECM to the cell [23,31]. Moreover, YAP is also involved in the transduction of morphogenetic signals of the Wnt pathway [23]. Recent studies have shown that components of the Hippo signaling pathway are involved in the fibrosis of various tissues, including the lungs [32], liver [33], kidneys [34] and heart [35]. Among them, the activation of the YAP/TAZ complex in interstitial myofibroblasts promotes fibrosis development in the kidneys [34].

Of note, type I collagen and hyaluronan (HA), together with its binding protein HABP2, are key ECM components of the fascia, as they promote fascial force transmission and fascial gliding, facilitating the smooth sliding of adjacent tissue layers [5]. Although a temporary increase in HA levels favors tissue fluidity, facilitating fascial gliding processes [5,36], a persistent increase in HA deposition leads to ECM stiffness [37]. Moreover, HABP2 can also induce tissue remodeling by inducing protease-activated receptors (PARs) signaling, which, in turn, promotes the activation of RhoA and Rho kinase (ROCK) [37] and of G proteins, which are known to regulate cell migration, proliferation, and the actin cytoskeleton’s physiology. YAP and TAZ are downstream effectors of the Rho-ROCK pathway [23]. Here, we showed that the gene expression levels of *COL1A* and *HABP2* were significantly increased after 2 h of treatment with the fESWs. Our data are in agreement with those reported by Ou W. et al. [38]. To investigate whether the YAP/TAZ complex modulates the expression of profibrotic genes, such as *ACTA2* and *COL1A1*, they utilized YAP/TAZ siRNA in cultured primary fibroblasts isolated from the intestines. They found that after YAP/TAZ knockdown, *ACTA2* and *COL1A1* were significantly down-regulated [38].

The knowledge of YAP’s role in fascia may explain the phenomenon of fibrosis after densification due to HA aggregation. Indeed, as reported by Stecco et al., fibrosis may occur as a direct long-term consequence of HA densification in some organs and tissues, such as fasciae [15]. Moreover, Schleip et al. observed that the density of fascial myofibroblasts (MFBs) in human lumbar fascia could be associated with an augmented occurrence of (micro-)injuries and related repair processes in the TLF [1]. Fibrogenesis is a mechanism of wound healing and repair, and a prolonged injury causes the deregulation of normal processes, resulting in an extensive deposition of ECM proteins and fibrosis [34,39,40]. MFB accumulation and excessive deposition of ECM components are common features in this stage of fibrosis.

However, some limitations are worth noting. This is the first study that investigated the expression of YAP in deep fascia; therefore, an analysis of deep fasciae collected from different topographical regions of the human body would give an even more complete understanding of the role of YAP in fascial biology. Nonetheless, this work constitutes the first step towards uncovering YAP’s influence on fascial remodeling, regeneration, fibrogenesis, and mechanotransduction.

## 4. Materials and Methods

### 4.1. Patients and Tissues

The patients included in this study underwent elective spine surgical procedures between March 2022 and October 2022 at the Orthopedic Clinic of the University of Padova. TLF tissues were obtained in the surgery room under sterile conditions from 8 patients (age range: 30–70 years; 4 males and 4 females) (Table 1). Exclusion criteria were malignant neoplasms, previous spine surgery, acute inflammatory disease, and infectious diseases. Tissue collection was approved by the Ethics Committee of the University Hospital of Padova (approval no. 3722/AO/16), and all patients gave their written informed consent.

All the samples were collected approximately 2–3 cm lateral to the lumbar 3 spinous process and were at least 2 cm × 2 cm. The specimens were divided into 2 parts: one was immediately snap-frozen and then stored in liquid nitrogen until protein extraction was performed. The second part was used for fascial fibroblast cell isolation.

### 4.2. Cell Isolation

Fibroblasts were isolated from the TLF of 4 patients (2 women and 2 men, patient n° 2, 3, 5, and 7). Fresh samples were collected in the surgery room in phosphate-buffered saline (PBS) containing 1% penicillin—streptomycin (Cod. #P0781, Sigma-Aldrich, Milan, Italy). The TLF was rinsed twice with PBS and then transferred to a Petri dish containing DMEM/F-12-GlutaMAX™ supplement (Cod#10565018, Gibco, Milan, Italy), supplemented with 10% FBS (Cod. # ECS0180L, Euroclone, Milan, Italy) and 1% penicillin—streptomycin (Cod. #P0781, Sigma-Aldrich, Milan, Italy). The specimens were minced into <1 mm × 1mm pieces in all dimensions and cultured at 37 °C and 5% CO_2_ until the fibroblasts were released from their native environment. After one week, the fibroblasts were observed in the cell culture. The small pieces of the TLF were gently removed, and the isolated cells were detached via trypsinization and centrifuged at 300× *g* for 5 min. After centrifugation, the cells were recovered, and the single-cell suspension was counted and transferred to a cell plate. The fibroblasts were cultured with a fresh culture medium that was replenished every 2–3 days.

### 4.3. Cell Characterization

To confirm that we isolated fibroblasts from the TLF tissues, primary cultures in early passages (passage 2 or 3) were assessed for the expression of the fibroblast markers CD90 and vimentin. Fibroblasts were seeded on coverslips at a density of 10,000 cells/slide and cultured for 48 h. The cells were fixed with 4% paraformaldehyde for 15 min, and were then permeabilized with 0.3% Triton X-100 (Cod # 93443, Sigma-Aldrich, Milan, Italy) in PBS for 10 min. The primary antibodies, namely mouse anti-vimentin (1:500, Cod #180052 Invitrogen) and mouse anti-CD90 (1:100, Cod. 66766-1-Ig Proteintech), diluted in 0.1% Triton PBS, were applied to the cells and incubated at 4 °C overnight, following which they were then incubated with a goat anti-mouse HRP-conjugate antibody (Cod #P026002 Agilent Dako, Santa Clara, CA, USA) at room temperature for 1 h. Positive immunostaining was detected with 3,3′–diaminobenzidine (Cod # K346711-2 Liquid DAB; Agilent Dako, Santa Clara, CA, USA); the reaction was developed for 1 min and stopped with water. Observations were carried out using a DM 2000 (Leica, Wetzlar, Germany) microscope. Negative control sections were prepared omitting the use of a primary antibody.

### 4.4. Cell Treatment with Focal Extracorporeal Shockwaves (fESWs)

Prior to carrying out the treatments, fibroblasts were seeded in 6-well plates at 3 × 10^4^ cells per well and grown to sub-confluence (80%). A focal ESW (fESW) treatment was performed, as previously described by Pirri et al., to mimic a mechanical stimulus [14]. The fESW treatment was administered using a Duolith SD-1 T-Top® device (Storz Medical, Tägewilen, Switzerland). This device employed an electromagnetic cylindrical coil as the source of focused shockwaves. In summary, each 6-multiwell plate containing cells was meticulously positioned so that the central point of the shockwave’s focal area precisely corresponded to the center of the bottom of the 6-multiwell plates. To achieve this level of accuracy, a bespoke 3D-printed support was designed for the ESW coil’s source, as previously described by Pirri et al. [14], ensuring that it maintained close contacts and perfect adherence to the shock unit alongside the 6-multiwell plate. Elaborate 3D computer-aided design (CAD) models for the support structure of the fESW cylindrical coil source were crafted using Autodesk Fusion 360 (Autodesk, Inc., San Rafael, CA, USA). The primary objective of this design process was to minimize the dissipation of fESW energy that typically occurs at the interface between the device’s head and the 6-multiwell plates. Subsequently, the CAD data were exported in STL format, making them compatible with 3D printing technology. A Ultimaker Creality Ender 3 printer was employed, operating at its highest resolution setting and utilizing polyvinyl chloride (PVC) as the printing material [10]. This support consists of two distinct parts: the upper component, which conforms to the size of the coil source, and the lower component, which serves a dual purpose of maintaining stability and suspending the support structure in relation to the 6-well plates. According to Pirri et al. [14], the cells were treated with 100 shots, a 2.5 Hz frequency, and an energy flux density of 0.25 mJ/mm^2^.

After fESW treatment, the fibroblasts were incubated for 30 min until protein extraction was performed, and for 2, 24, and 48 h until total RNA extraction was performed.

### 4.5. Immunoblotting

Total proteins were extracted in RIPA lysis buffer (Cod #89900, Thermo Scientific, Milan, Italy) containing protease inhibitors (Cod. #C0001, Protease Inhibitor Cocktail, TargetMol, Boston, MA) and phosphatase inhibitors (Cod #C0002, Phosphatase Inhibitor Cocktail I, TargetMol, Boston, MA), and protein concentration was measured with a Pierce™ BCA Protein Assay Kit (Cat # 23227, Thermo Scientific, Milan, Italy). Proteins (20 µg) were separated in an 8% polyacrylamide gel and electro-blotted onto a nitrocellulose membrane (Cod. #10600008, Amersham-Hybond EC, GE Healthcare Life Sciences, Milan, Italy). The membranes were blocked for 1h at room temperature in 5% non-fat dry blocking milk and then incubated overnight at 4 °C with the primary antibodies against YAP (1:1000; sc-101199, Santa Cruz, Dallas, TX, USA) and a polyclonal antibody against Phospho-YAP (Ser 127; 1:1000; Cod #4911, Cell Signaling, Danvers, MA, USA). After washing, the membranes were incubated for 1 h with a horseradish peroxidase-labeled secondary antibody and visualized via chemiluminescence using the Super Signal West Pico Chemiluminescent Substrate (Cod # 34577, Thermo Scientific, Milan, Italy). Band intensity was measured in ATOM UVITEC (Uvitec, Milan, Italy). Images were analyzed with the Nine Alliance Program (Uvitec, Milan, Italy). To adjust for differences in the amount of loaded protein, YAP and p-YAP expression levels were normalized to ®-actin (1:5000, Cod. #A5441, Sigma-Aldrich, Milan, Italy).

### 4.6. RNA Extraction and Real-Time PCR

Total RNA was isolated from fibroblasts using a High pure RNA isolation Kit (Cod. #11828665001, Roche Diagnostics, Milan, Italy), following the manufacturer’s protocol. One µg of total RNA was then reverse-transcribed with Iscript (Cod. # 1708841, Bio-Rad Laboratories, Milan, Italy) in a final volume of 20 µL. We used 100 ng of cDNA to perform real-time RT-PCR using 4X CAPITAL qPCR Green Master Mix (Cod. #BR0501702, Biotechrabbit, Berlin, Germany). PCR was performed using the CFX96™ instrument (Bio-Rad Laboratories, Milan, Italy) with the following protocol: 95 °C for 3 min, followed by 50 cycles of 95 °C for 15 s, and 60 °C for 30 s. The relative expression levels of collagen 1 (*COL1A1*) and hyaluronan-binding protein 2 (*HABP2*) mRNA were measured with real-time RT-PCR and calculated using the comparative Ct (2^−ΔΔCt^) method, using *GAPDH* as a housekeeping gene. Primers were designed using Pimer3 Software ver. 4.1 and are reported in Table 2.

**Table 1:** Patient’s descriptive data.

Patient Number	Age	Sex
1	30	F
2	37	F
3	45	M
4	45	F
5	54	F
6	61	M
7	68	M
8	70	M

**Table 2:** Primers used for gene expression analysis with real-time PCR.

Gene (Accession Number)	Forward Primer	Reverse Primer
***COL1A1*** **NM_000088.4**	5′- TTCTCAGCGTGGGTAAGTGT- 3′	3′- TTCTCAGCGTGGGTAAGTGT- 5′
***HABP2*** **NM_001177660.3**	5′- AATGGCTCTGGTGGGAAAGA- 3′	3′- TTCTCAGCGTGGGTAAGTGT- 5′
***GAPDH*** **NM_001256799.3**	5′- TTCTCAGCGTGGGTAAGTGT- 3′	3′- TTCTCAGCGTGGGTAAGTGT- 5′

### 4.7. The Cell Proliferation Assay

Fascial fibroblasts were seeded at an initial cell density of 20 × 10^5^ cells per well in a 6-well plate and cultured until reaching 60% confluence. Cells were pre-treated with 5 μM verteporfin (Cod. #SML0534, Sigma-Aldrich, Milan, Italy), a well-known inhibitor of YAP activity [30], for 1 h and then stimulated with fEWSs. After the fEWS treatment, fibroblasts were placed in the IncuCyte S3 Live-Cell Analysis System (Sartorius, Surrey, UK) to record the proliferation rate over time. Time-lapse microscopy images were captured every 4 h for 16 h, for a total of 121 images per well for each time point using a 10× objective. Applying an adherent cell-by-cell analysis, values of cell count/mm^2^ were obtained from each image (mean ± SEM). Data were pooled for the single well and averaged across the 4 patients (n° 2, 3, 5, and 7), and were normalized to time 0.

### 4.8. Statistical Analysis

Statistical analysis was performed using GraphPad 8.4.2. (GraphPad software Inc., San Diego, CA, USA), and a *p* < 0.05 was considered as a limit for statistical significance. The normality assessment was carried out using the Kolmogorov–Smirnov test. All results are presented as the mean ± standard deviation or standard error, as reported in each figure legend. Differences between the total and inactive YAP forms, and gene expression levels between the treated and untreated cells were analyzed using the Mann–Whitney test. Differences between the proliferation curves among different treatments were analyzed using the 2-way ANOVA and Tukey’s multiple comparisons tests.

## 5. Conclusions

These findings demonstrate that YAP is present in the TLF and can be considered a “new player” in the mechanobiology of deep fascia, suggesting its involvement in the fascial remodeling, regeneration, fibrogenesis, and mechanotransduction processes. We are sure that, with integrated research efforts, we will better understand the YAP pathway in fascial tissue under physiological and pathological conditions and, therefore, identify new targets for drug treatments.

## Data Availability

The data presented in this study are available on request from the corresponding author. The data are not publicly available due to privacy.

## References

[1] Schleip R., Gabbiani G., Wilke J., Naylor I., Hinz B., Zorn A., Jäger H., Breul R., Schreiner S., Klingler W. (2019). Fascia Is Able to Actively Contract and May Thereby Influence Musculoskeletal Dynamics: A Histochemical and Mechanographic Investigation. Front. Physiol..

[2] Mense S. (2019). Innervation of the thoracolumbar fascia. Eur. J. Transl. Myol..

[3] Hoheisel U., Rosner J., Mense S. (2015). Innervation changes induced by inflammation of the rat thoracolumbar fascia. Neuroscience.

[4] Kondrup F., Gaudreault N., Venne G. (2022). The deep fascia and its role in chronic pain and pathological conditions: A review. Clin Anat..

[5] Fede C., Pirri C., Fan C., Petrelli L., Guidolin D., De Caro R., Stecco C. (2021). A Closer Look at the Cellular and Molecular Components of the Deep/Muscular Fasciae. Int. J. Mol. Sci..

[6] Panciera T., Azzolin L., Cordenonsi M., Piccolo S. (2017). Mechanobiology of YAP and TAZ in Physiology and Disease. Nat. Rev. Mol. Cell Biol..

[7] Langevin H.M., Cornbrooks C.J., Taatjes D.J. (2004). Fibroblasts Form a Body-Wide Cellular Network. Histochem. Cell Biol..

[8] Theocharis A.D., Skandalis S.S., Gialeli C., Karamanos N.K. (2016). Extracellular Matrix Structure. Adv. Drug Deliv. Rev..

[9] Benjamin M. (2009). The Fascia of the Limbs and Back—A Review. J. Anat..

[10] Lendahl U., Muhl L., Betsholtz C. (2022). Identification, Discrimination and Heterogeneity of Fibroblasts. Nat. Commun..

[11] Wang T., Long Y., Ma L., Dong Q., Li Y., Guo J., Jin L., Di L., Zhang Y., Wang L. (2023). Single-Cell RNA-Seq Reveals Cellular Heterogeneity from Deep Fascia in Patients with Acute Compartment Syndrome. Front. Immunol..

[12] Jansen K.A., Donato D.M., Balcioglu H.E., Schmidt T., Danen E.H.J., Koenderink G.H. (2015). A Guide to Mechanobiology: Where Biology and Physics Meet. Biochim. Biophys. Acta-Mol. Cell Res..

[13] Frairia R., Berta L. (2011). Biological Effects of Extracorporeal Shock Waves on Fibroblasts. A Review. Muscles. Ligaments Tendons J..

[14] Pirri C., Fede C., Petrelli L., De Rose E., Biz C., Guidolin D., De Caro R., Stecco C. (2022). Immediate Effects of Extracorporeal Shock Wave Therapy in Fascial Fibroblasts: An In Vitro Study. Biomedicines.

[15] Fede C., Pirri C., Petrelli L., Guidolin D., Fan C., De Caro R., Stecco C. (2020). Sensitivity of the Fasciae to the Endocannabinoid System: Production of Hyaluronan-Rich Vesicles and Potential Peripheral Effects of Cannabinoids in Fascial Tissue. Int. J. Mol. Sci..

[16] Fede C., Pirri C., Fan C., Albertin G., Porzionato A., Macchi V., De Caro R., Stecco C. (2019). Sensitivity of the Fasciae to Sex Hormone Levels: Modulation of Collagen-I, Collagen-III and Fibrillin Production. PLoS ONE.

[17] Pirri C., Caroccia B., Angelini A., Petrelli L., Piazza M., Biz C., Ruggieri P., De Caro R., Stecco C. (2022). Evidence of Renin–Angiotensin System Receptors in Deep Fascia: A Role in Extracellular Matrix Remodeling and Fibrogenesis?. Biomedicines.

[18] Stecco A., Cowman M., Pirri N., Raghavan P., Pirri C. (2022). Densification: Hyaluronan Aggregation in Different Human Organs. Bioengineering.

[19] Zanconato F., Forcato M., Battilana G., Azzolin L., Quaranta E., Bodega B., Rosato A., Bicciato S., Cordenonsi M., Piccolo S. (2015). Genome-Wide Association between YAP/TAZ/TEAD and AP-1 at Enhancers Drives Oncogenic Growth. Nat. Cell Biol..

[20] Dupont S., Morsut L., Aragona M., Enzo E., Giulitti S., Cordenonsi M., Zanconato F., Le Digabel J., Forcato M., Bicciato S. (2011). Role of YAP/TAZ in Mechanotransduction. Nature.

[21] Gaspar P., Tapon N. (2014). Sensing the Local Environment: Actin Architecture and Hippo Signalling. Curr. Opin. Cell Biol..

[22] Nakajima H., Yamamoto K., Agarwala S., Terai K., Fukui H., Fukuhara S., Ando K., Miyazaki T., Yokota Y., Schmelzer E. (2017). Flow-Dependent Endothelial YAP Regulation Contributes to Vessel Maintenance. Dev. Cell.

[23] Piccolo S., Dupont S., Cordenonsi M. (2014). The Biology of YAP/TAZ: Hippo Signaling and Beyond. Physiol. Rev..

[24] Rege T.A., Hagood J.S. (2006). Thy-1 as a Regulator of Cell-cell and Cell-matrix Interactions in Axon Regeneration, Apoptosis, Adhesion, Migration, Cancer, and Fibrosis. FASEB J..

[25] Cheng F., Shen Y., Mohanasundaram P., Lindström M., Ivaska J., Ny T., Erikss J.E. (2016). Vimentin Coordinates Fibroblast Proliferation and Keratinocyte Differentiation in Wound Healing via TGF-β-Slug Signaling. Proc. Natl. Acad. Sci. USA.

[26] Calvo F., Ege N., Grande-Garcia A., Hooper S., Jenkins R.P., Chaudhry S.I., Harrington K., Williamson P., Moeendarbary E., Charras G. (2013). Mechanotransduction and YAP-Dependent Matrix Remodelling Is Required for the Generation and Maintenance of Cancer-Associated Fibroblasts. Nat. Cell Biol..

[27] Halder G., Dupont S., Piccolo S. (2012). Transduction of Mechanical and Cytoskeletal Cues by YAP and TAZ. Nat. Rev. Mol. Cell Biol..

[28] Lorthongpanich C., Thumanu K., Tangkiettrakul K., Jiamvoraphong N., Laowtammathron C., Damkham N., U-Pratya Y., Issaragrisil S. (2019). YAP as a Key Regulator of Adipo-Osteogenic Differentiation in Human MSCs. Stem Cell Res. Ther..

[29] Liu-Chittenden Y., Huang B., Shim J.S., Chen Q., Lee S.J., Anders R.A., Liu J.O., Pan D. (2012). Genetic and Pharmacological Disruption of the TEAD-YAP Complex Suppresses the Oncogenic Activity of YAP. Genes Dev..

[30] Wang C., Zhu X., Feng W., Yu Y., Jeong K., Guo W., Lu Y., Mills G.B. (2016). Verteporfin Inhibits YAP Function through Up-Regulating 14-3-3σ Sequestering YAP in the Cytoplasm. Am. J. Cancer Res..

[31] Pan D. (2010). The Hippo Signaling Pathway in Development and Cancer. Dev. Cell.

[32] Link P.A., Choi K.M., Diaz Espinosa A.M., Jones D.L., Gao A.Y., Haak A.J., Tschumperlin D.J. (2022). Combined Control of the Fibroblast Contractile Program by YAP and TAZ. Am. J. Physiol.-Lung Cell. Mol. Physiol..

[33] Mooring M., Fowl B.H., Lum S.Z., Liu Y., Yao K., Softic S., Kirchner R., Bernstein A., Singhi A.D., Jay D.G. (2020). Hepatocyte Stress Increases Expression of Yes-Associated Protein and Transcriptional Coactivator with PDZ-Binding Motif in Hepatocytes to Promote Parenchymal Inflammation and Fibrosis. Hepatology.

[34] Liang M., Yu M., Xia R., Song K., Wang J., Luo J., Chen G., Cheng J. (2017). Yap/Taz Deletion in Gli^+^ Cell-Derived Myofibroblasts Attenuates Fibrosis. J. Am. Soc. Nephrol..

[35] Mia M.M., Cibi D.M., Ghani S.A.B.A., Singh A., Tee N., Sivakumar V., Bogireddi H., Cook S.A., Mao J., Singh M.K. (2022). Loss of Yap/Taz in Cardiac Fibroblasts Attenuates Adverse Remodelling and Improves Cardiac Function. Cardiovasc. Res..

[36] Wilke J., Niederer D., Vogt L., Banzer W. (2016). Remote effects of lower limb stretching: Preliminary evidence for myofascial connectivity?. J. Sports Sci..

[37] Mambetsariev N., Mirzapoiazova T., Mambetsariev B., Sammani S., Lennon F.E., Garcia J.G.N., Singleton P.A. (2010). Hyaluronic Acid Binding Protein 2 Is a Novel Regulator of Vascular Integrity. Arterioscler. Thromb. Vasc. Biol..

[38] Ou W., Xu W., Liu F., Guo Y., Huang Z., Feng T., Liu C.Y., Du P. (2021). Increased Expression of Yes-Associated Protein/YAP and Transcriptional Coactivator with PDZ-Binding Motif/TAZ Activates Intestinal Fibroblasts to Promote Intestinal Obstruction in Crohn’s Disease. eBioMedicine.

[39] Kisseleva T., Brenner D.A. (2008). Mechanisms of fibrogenesis. Exp. Biol. Med..

[40] Dasgupta I., McCollum D. (2019). Control of Cellular Responses to Mechanical Cues through YAP/TAZ Regulation. J. Biol. Chem..

